# Targeted modification of *CmACO1* by CRISPR/Cas9 extends the shelf-life of *Cucumis melo* var. *reticulatus* melon

**DOI:** 10.3389/fgeed.2023.1176125

**Published:** 2023-05-25

**Authors:** Satoko Nonaka, Maki Ito, Hiroshi Ezura

**Affiliations:** ^1^ Tskuba Plant Innovation Research Center, University of Tsukuba, Tsukuba, Ibaraki, Japan; ^2^ Department of Agricultural Sciences, Institute of Life and Environmental Sciences, University of Tsukuba, Tsukuba, Ibaraki, Japan; ^3^ College of Agro-Biological Resources, University of Tsukuba, Tsukuba, Ibaraki, Japan

**Keywords:** melon, genome editing, CRISPR/Cas9, shelf-life, ethylene, ACC oxidase

## Abstract

The gaseous plant hormone ethylene is a regulator of fruit shelf-life, one of the essential traits in fruits. Extending fruit shelf-life reduces food loss, thereby expected to contribute to food security. The enzyme 1-aminocyclopropane-1-carboxylic acid oxidase (ACO) is the final step of the ethylene production pathway. Its suppression via antisense technology has been demonstrated to extend the shelf-life of melon, apple, and papaya. Genome editing technology is an innovative technique for plant breeding. Because the genome editing technology would not leave the exogenous genes in the final crop products, the crops via genome editing can be considered non-genetically modified yields; compared to conventional breeding, such as mutation breeding, the breeding term would be expected to be relatively short. These points include the advantage of this technique in utilization for commercial applications. We attempted to extend the shelf-life of the Japanese luxury melon (*Cucumis melo* var. reticulatus, ‘Harukei-3’) via modification of the ethylene synthesis pathway with the genome editing technology, CRISPR/Cas9 system. The Melonet-DB (https://melonet-db.dna.affrc.go.jp/ap/top) showed that the melon genome had the five *CmACOs* and the gene *CmACO1* predominantly expressed in harvested fruits. From this information, *CmACO1* was expected to be a key gene for shelf-life in melons. Based on this information, the *CmACO1* was selected as the target of the CRISPR/Cas9 system and introduced the mutation. The final product of this melon did not have any exogenous genes. The mutation was inherited for at least two generations. In the T_2_ generation, the fruit phenotypes 14 days after harvest were as follows: ethylene production was reduced to one-tenth that of the wild type, pericarp colour remained green, and higher fruit firmness. Early fermentation of the fresh fruit was observed in the wild-type fruit but not in the mutant. These results show that *CmACO1* knockout via CRISPR/Cas9 extended the melon’s shelf-life. Moreover, our results suggest that genome editing technology would reduce food loss and contribute to food security.

## 1 Introduction

Long shelf-life is a crucial trait in fruit crop breeding since it directly affects food loss and waste. Globally, 14% of total food production is lost before retail sales (FAO, 2019), and an additional 17% is wasted at the retail and consumer levels (United Nations Environment Programme, 2021), resulting in a staggering loss of USD $400 billion. These losses are especially significant for fruits and vegetables due to their poor shelf-life compared to cereals and non-perishable goods (FAO, 2019). Therefore, the ability to modify plants to improve shelf-life is expected to improve the sustainability of the global food system by reducing food loss and waste. Moreover, having a long shelf-life is crucial for exporting fruits and vegetables, as maintaining fruit quality and freshness requires close control of temperature, humidity, oxygen, and carbon dioxide, leading to high energy usage and costs. Therefore, producing a parental line that promotes a long shelf-life can reduce the energy and costs required for export (Lamberty and Kreyenschmidt, 2022). Genome-edited crops have already been commercialized in Japan and the United States, proving their usefulness as a breeding tool.

Fruits are categorized into “climacteric” and “non-climacteric,” depending on the ripening style. The shelf-life in climacteric fruits is controlled by the gaseous phytohormone ethylene, synthesized from the amino acid methionine and first converted to S-adenosyl-L-methionine (SAM) by SAM synthase. SAM is the primary methyl donor in plants and is involved in the methylation of lipids, proteins, and nucleic acids. SAM is converted by the enzyme aminocyclopropanecarboxylate (ACC) synthase to 5′-methylthioadenosine, which is converted back to methionine and then to ACC, the precursor of ethylene. ACC is finally oxidized by ACC oxidase (ACO) to form ethylene. Therefore, ACO is a key enzyme in the regulation of ethylene production. In many plant species, the *ACO* gene has several homologs with highly conserved sequences in the genes. The amino acid sequence of ACO has an RXS motif in its C-terminal. Depending on the RXS motif, ACOs are classified into Type I: RMS, Type II: RL/IS, and Type III: RRS (Houben and Van de Poel, 2019).

Reducing the enzymatic activity in climacteric fruit can extend their postharvest shelf-life. Previous studies have shown that suppressing the *ACO* gene via antisense or RNA-i genetic modification extends fruit shelf-life by 5–14 days in climacteric fruits such as apple, papaya, kiwi, tomato, and melon (Ayub et al., 1996; Nuñez-Palenius et al., 2006; López-Gómez et al., 2009; Batra et al., 2010; Atkinson et al., 2011; Sekeli et al., 2014). Substitutional mutation of *ACO* via ethyl methanesulfonate (EMS) treatment and Targeting Induced Local Lesions in Genomes (TILLING) selection also extended the shelf-life in melon (Dahmani-Mardas et al., 2010). The TILLIG study showed the mutation of G194D resulted in a longer shelf-life in melon fruit (Dahmani-Mardas et al., 2010).

The antisense and RNAi techniques are based on the transformant technique, and crops created by this technique are categorized as genetically modified crops (GM-crops). G.M. crops are heavily regulated and require costly safety evaluation. Furthermore, they tend to receive poor public opinion. Although mutation induction via EMS treatment has been utilised as a breeding technology, the technique is slow, requiring extended periods to introduce new, valuable traits into elite breeding parent. Furthermore, it is unsuitable to use in vegetatively propagated crops because, during the backcrossing, the genome set of the original will be lost. To avoid these problems, new techniques for genetic manipulation are needed.

Genome editing technologies present a promising solution because they can directly modify the target genes of elite strains and produce a final line without any exogenous genes. Technologies such as zinc finger nucleases, transcription activator-like effector nucleases (TALENs), and clustered, regularly interspaced, short palindromic repeats (CRISPR)/CRISPR-associated protein 9 (CRISPR/Cas9) enable the direct introduction of mutations into the target genes. These nucleases induce double-strand breaks (DSBs) at a specific genome region, which are then repaired. For the repair of DSBs in plants, the non-homologous end-joining (NHEJ) pathway is preferentially utilized. The NHEJ pathway is relatively error-prone, so the mutation is directly induced in the DSB site. This genome editing technology enables mutation induction by defusing the genetic function of the target gene (Ito et al., 2015; Giordano et al., 2022) or deleting a domain to modify function (Nonaka et al., 2017). Genome editing technologies have been utilized to analyze gene function and introduce valuable traits into crops. For example, the use of CRISPR/Cas9 to produce tomatoes with high gamma-aminobutyric acid content and of TALENs to raise the oleic acid content of soybeans has already been commercialized in Japan and the United States (Ku and Ha, 2020). Among the genome editing technologies, CRISPR/Cas9 yields the highest mutation frequency (Jinek et al., 2012) and requires only a 20 bp target sequence in the genome region before the protospacer-adjacent motif (PAM) sequence (5′-NGG for the type II CRISPR/Cas9 system). Furthermore, compared to conventional breeding methods such as cross and mutagenesis, utilization of the CRISPR/Cas9 system enables short-term breeding.

In this study, we aimed to introduce a mutation to the ACC oxidase gene, *ACO*, using the CRISPR/Cas9 system to extend shelf-life in the climacteric type melon “Earl’s favourite Harukei-3”, which is used as a parental line for breeding exclusive Japanese melon cultivars that represent important export crops. The results of this study could contribute to the Agricultural field in the following three ways: 1) improving shelf-life, which helps farmers economically and reduces food waste; 2) providing a method for plant breeders to quickly and efficiently produce desired mutations in crops; and 3) producing non-GM crops with desirable traits, circumventing pitfalls associated with G.M. produce.

## 2 Methods and materials

### 2.1 *in silico* analysis

The Melonomics v4.0 database (https://www.melonomics.net/melonomics.html; Ruggieri et al., 2018) was used for gene analysis. The ‘Gene expression map viewer’ and ‘Co-expression viewer’ in Melonet-DB (https://melonet-db.dna.affrc.go.jp/ap/top; Yano et al., 2018; Yano et al., 2020) were used to check gene expression levels in melon organs. Geneious Prime (Alignment type, global alignment with free end gaps; cost matrix, Blosum62; genetic distance model, Jukes-Cantor; tree build method, neighbor-joining; Java Version 11.0.14.1 + 1 [64 bit]) was used for the comparison of aminoacid sequence of ACOs. The target sites were selected by CRISPR-P (http://crispr.hzau.edu.cn/CRISPR2/). These sites represented potential genome-wide off-target sites and were identified using the CRISPR RGEN Tools website (http://www.rgenome.net/cas-offinder/). The candidate off-target sites were not found in Cas-OFFinder.

### 2.2 Plant materials and growth conditions

Seeds of the cultivated melon *Cucumis melo* var. *reticulatus* (accession “Earl’s favourite Harukei-3”) were obtained from the GenBank of the National Agriculture and Food Research Organization (NARO) in Japan. Seeds were germinated in soil under dark conditions at 25°C–28°C in March or April. Seedlings were grown in an NAE Terrace production system (Mitsubishi Chemical Agri Dream Co. Ltd., Tokyo, Japan) under a 9 h light (25°C) and 15 h dark (20°C) cycle until true leaf expansion was observed. Plants were then transferred to soil composed of ‘Coco-bag’ (Toyotane Co. Ltd., Toyohashi, Japan) and were grown under greenhouse conditions. Plants were irrigated with Otsuka standard nutrient solution (OAT Agrio Co. Ltd., Tokyo, Japan) at an electrical conductivity of 1.2–2.4 dS/m. Female flowers were hand pollinated with pollen from male anthers obtained from the same plant (self-pollination), such that one or two fruits developed on each plant. In the case of disease emergence (powdery mildew, whitefly, canker, etc.), chemicals were applied to the plant. A quantum sufficing of commercial fungicide was sprayed according to the attached instructions. For example, Pancho T.F. granule wettable powder (Nippon Soda Co. Ltd. https://www.nippon-soda.co.jp/e/fields_and_products/) was diluted 1,000 times and used.

Transgenic shoots of the T_0_ generation were acclimated in a growth room at 25°C under 16/8-h light/dark conditions. Once these plants produced their 10th leaf, they were transferred to the Special Netted-house at the University of Tsukuba, Japan. The T_0_ generation was cultivated in containers with potting soil (Genki-kun 1 gou, Katakura & Co-op Agri Corporation; http://www.katakuraco-op.com/site_fertilizer/products/pdf/genkikun_1gou.pdf, Tokyo, Japan) and watered twice daily.

Transgenic seeds of the T_1_ and T_2_ generations and control wild type (W.T.) seeds were germinated on wet filter paper and then transferred to rockwool 2 weeks after germination. Seedlings were cultured in a growth room described above for 1 month until five to six true leaves emerged and then transferred to the Special Netted-house at the University of Tsukuba. All plants were fertilized using a nutrient film technique cultivation system and irrigated with Otsuka standard nutrient solution at an electrical conductivity of 1.2–2.4 dS/m. Cultivation for this experiment was carried out from April to August 2018. Each plant produced one fruit, which was harvested 50 days after pollination.

The generation of transgenic shoots regenerated from calli is denoted as T_0_ generation. The following generations are denoted as T_1_, T_2_, and T_3_.

### 2.3 Vector construction and melon transformation

The CRISPR/Cas9 system was introduced into the melon genome via Agrobacterium-mediated transformation, following the protocol established by Akasaka-Kennedy et al. (2004). The vectors used for modification with the CRISPR/Cas9 system (pDeCas9_Kan) were kindly provided by Dr. Masaki Endo (NARO, Japan). The pDeCas9 vector contains the following parts: L.B. (left border sequence), Pubi (ubiquitin promoter form parsley), Cas9 (derived from *Streptococcus pyogenes*), Tpea3A (heat shock protein 17.3 kDa terminator from *Glycine max*), PAtu6 (U6-26 promoter, or RNA polymerase III promoter, from *Arabidopsis thaliana*), Pnos (nopaline synthase gene promoter from *Agrobacterium tumefaciens*), Tnos (nopaline synthase gene terminator from *A. tumefaciens*), R.B. (right border), nptII (neomycin phosphotransferase, or kanamycin resistance gene), and guide RNA. These plasmids were modified following methods described in a previous study (Mikami et al., 2015). Following the instrument manual of Gene Pulser Xcell Electroporation System, the constructed vectors were introduced into *A. tumefaciens* GV2260 (Deblaere et al., 1985) via electroporation (Gene Pulser Xcell Electroporation Systems, Bio Rad Laboratories, Inc., CA, United States).

### 2.4 Ploidy analysis

The ploidy of the rooting shoots was determined using flow cytometry. One square centimeter of leaf tissue was cut from the rooting shoots, then chopped and added to 250 µL of nucleus-extraction solution (CyStain UV Precise P, Sysmex Corporation, Kobe, Japan). The solution was filtered through 1 mm^2^ mesh, after which 1 mL of staining solution (CyStain UV Precise P, Sysmex Corporation) was added, and the solution was incubated for 1 min. The stained solution was then analyzed using a Quantum P (Cytotechs, Ibaraki, Japan) to identify 2n plants. The 2n plants were planted on mineral wool (Grodan, Roermond, Netherlands) and acclimatized in a growth room under 25°C and a 16/8 h light/dark cycle.

### 2.5 Southern blot analysis

Genomic DNA was extracted from young melon leaves using Maxwell 16 System DNA Purification kits (Promega Corporation, Madison, WI, United States). The purified DNA was then digested with *Xba*I, electrophoretically separated on a 0.8% agarose gel with λ/*Hin*dIII as a marker, and transferred to Gene Screen Plus nylon membranes (Roche Diagnostics Ltd., Basel, Switzerland) using 20X saline-sodium citrate buffer. After ultraviolet cross-linking, the membranes were hybridized in a solution containing 7% sodium dodecyl sulfate, 50% deionized formamide, 50 mM sodium phosphate (pH 7.0), 2% blocking solution, 0.1% N-lauroylsarcosine, 0.75 M NaCl, and 75 mM sodium citrate at 42 °C overnight. For hybridization, a digoxigenin (DIG)-labeled DNA probe specific to *nptII* (0.8 Kb) was generated through polymerase chain reaction (PCR) with the following primer set: nptII_Fw: 5′- ATG​ATT​GAA​CAA​GAT​GGA​TTG​C-3′ and nptII_Rv: 5′-TCA​GAA​GAA​CTC​GTC​AAG​AAG​G-3′, and the DIG-High Prime DNA Labeling Kit (Roche Diagnostics Ltd., Basel, Switzerland). The DIG signal was detected by a Detection Starter Kit (Roche Diagnostics Ltd., Basel, Switzerland) and Luminescent Image Analyzer Las-1000 (FUJIFILM Corporation, Tokyo, Japan), following the manufacturer’s protocol.

### 2.6 Genetic analysis

To confirm the sequence of the target site, a 1,134 bp genomic region was amplified using a primer set (ACO1741F2: 5′-ACC​CAC​CAA​CCA​AAA​ACA​A-3′ and ACO11874R1: 5′-GTT​AAC​ACT​CTA​TGC​A-3′) and DNA polymerase KOD Plus Neo (Toyobo Co. Ltd., Osaka, Japan). The amplified fragment was purified using ExoSAP-IT reagent (Thermo Fisher Scientific Inc., Waltham, MA, United States). The purified fragment was inserted into the pGEM^®^-T Easy Vector system (Promega Corporation, Madison, WI, United States). The cloning plasmid was introduced into *Escherichia coli* JM109 competent cells, Compitent high JM109 (Toyobo Co. Ltd., Osaka, Japan). The cells were plated onto a lysogeny broth medium with 100 mg/L of ampicillin (FUJIFILM Wako Pure Chemical Corp., Tokyo, Japan). For blue and white selection, 0.2 mg/L of 5-bromo-4-chloro-3-indolyl-β-D-galactopyranoside (X-gal, Wako) and 1 mM isopropyl β-D-1-thiogalactopyranoside (FUJIFILM Wako Pure Chemical Corp., Tokyo, Japan) were added. Blue colonies indicate failed cloning, while white colonies indicate successful cloning into the pGEM^®^-T Easy Vector. The details of the blue and white selections were found in the instrument manual of the pGEM^®^-T Easy Vector system. For the white colony, the insertion was verified by colony PCR using SP6 and T7 primer sets and GoTaq^®^ DNA polymerase (Promega Corporation, Madison, WI, United States). The plasmid was extracted from the white colony with an insert using a QIAprep Spin Miniprep Kit (Qiagen, Venlo, Netherlands). The clones were analyzed by Sanger sequencing with the SP6 promoter, and the resulting nucleic acid sequence was translated to the corresponding amino acid sequence.

### 2.7 Firmness

The firmness of the melon fruit was determined by cutting it into pieces and using a Brookfield CT3 Texture Analyzer (AMETEK Brookfield, Middleborough, MA, United States; https://www.brookfieldengineering.com/products/texture-analyzers/ct3-texture-analyzer) with a TA3/100 probe (25.4 mm in diameter, 35 mm in length). The maximum downward vertical force into the fresh fruit (with a trigger speed of 0.2 mm s^–1^ and load 5.0 g) was recorded with the texture analyzer. Three experimental replicates were performed for each fruit sample.

### 2.8 ACC oxidase activity

We used younger leaves to detect ethylene evolution. Shoot apicals were placed in 50 mL vials containing 35 mL of Murashige Skoog’s medium (M.S.), with a pH of 5.8, 3% sucrose, and 1% agarose (FUJIFILM Wako Pure Chemical Corp., Tokyo, Japan) (Murashige and Skoog, 1962) to create a gas space of 15 mL. To observe the ACO activity, 200 µM of ACC (Wako) was added to the M.*S. medium* and incubated at 30 °C for 24 h under light/dark 16/8 h conditions. M.*S. medium* with 0 µM of ACC was also prepared as a control. After incubation, 1 mL of the air phase in the vials was subjected to gas chromatography using a stainless-steel column packed with activated alumina, heated to 50°C, and flame-ionization detection at 120°C (GC14-B; Shimadzu Corporation, Kyoto, Japan). The methodology is described in Supplementary Figure S1.

### 2.9 Apical ethephon treatment

We sprayed 1% of ethephon (Nissan Ethrel 10, Nissan Chemical Corporation, Tokyo, Japan) on the shoot apexes. After spraying, the apexes were covered with a Tedlar gas sampling bag (polyvinyl fluoride, G.L. Science Inc., Tokyo, Japan) for 7 days.

### 2.10 Ethylene measurement from fruit

Ethylene evolution was analyzed using an SGEA-P3-C1 ethylene detection sensor system (Nissha, Kyoto, Japan). A polypropylene airtight container with a capacity of 0.98 L was connected to the measuring instrument using a fluororesin tube with an inner diameter of 3 mm. Fruits were placed in a container and sealed to measure the amount of ethylene produced every 15 min for 8 h. The average rate of ethylene generation during the measurement period was calculated.

### 2.11 Evaluation of the fruit shelf-life

To assess the shelf-life of the melons, fruits were harvested 50 days after pollination and stored at 25°C for 14 days in dark conditions. Ethylene evolution was measured using the method described in Section 2.10.

## 3 Results

### 3.1 Selection of the target gene and site for CRISPR/Cas9

**FIGURE 1:**
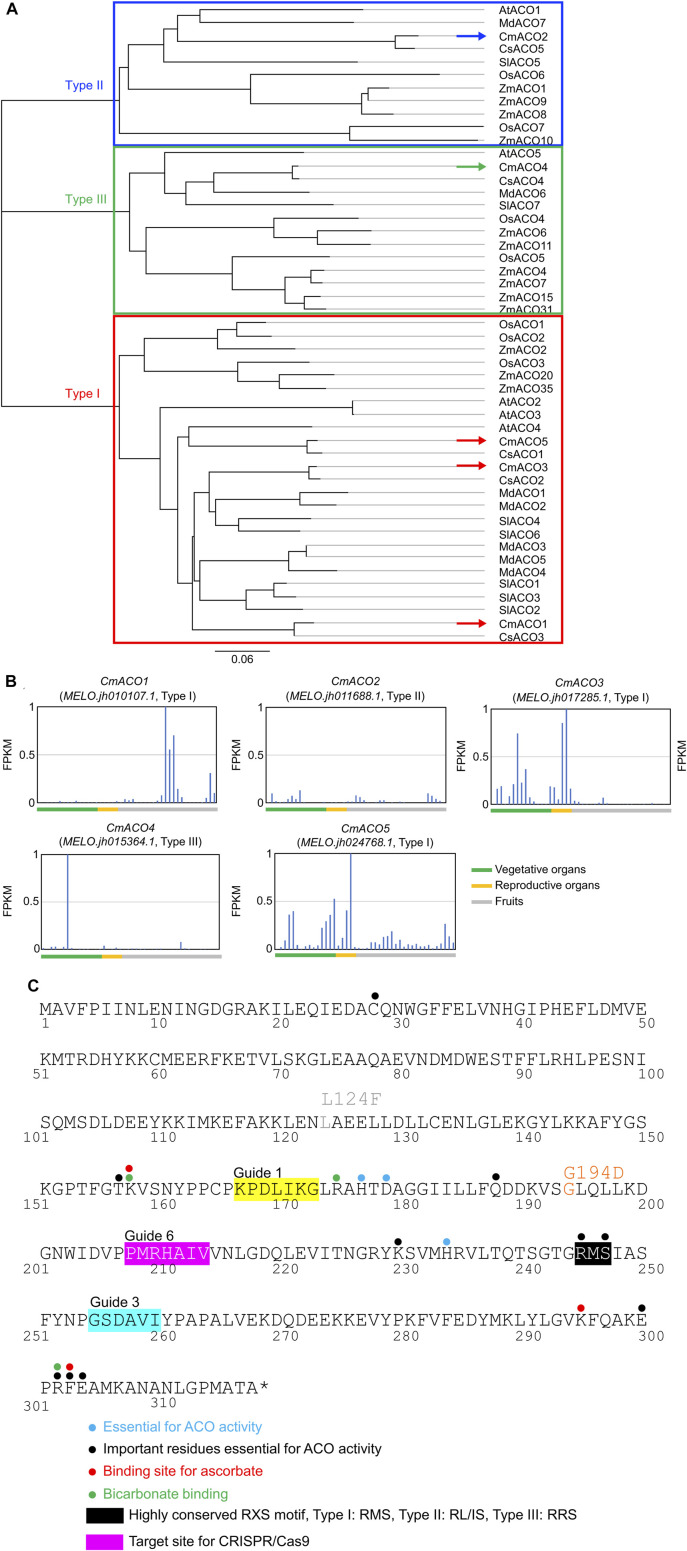
Characteristics of *CmACOs* in amino acid sequences and gene expression patterns **(A)** Phylogenic tree of ACO amino acid sequence in agriculturally important. **(B)** *CmACO* gene expression pattern in each plant organ. **(C)** Amino acid sequence of highly conserved motif and target sites for CRISPR/Cas9 in *CmACO1*. Based on the apple experiments (Dilley et al., 2013), blue, black, red, and green dots represent essential sites for ACO activity, important residues essential for ACO activity, ascorbate binding, and bicarbonate binding, respectively. Black and pink boxes indicated the highly conserved RXS motif and the target site for CRISPR/Cas9, respectively.

Five *CmACO* (*CmACO1–5*) genes were found in the Melonomics v4.0 database and Melonet-DB. *CmACO1–3* represented previously identified sequences (Lasserre et al., 1996), and *CmACO4–5* were identified as new genes. Gene IDs were MELO.jh010107.1 (*CmACO1*), MELO.jh011688.1 (*CmACO2*), MELO.jh017285.1 (*CmACO3*), MELO.jh015364.1 (*CmACO4*), and MELO.jh024768.1 (*CmACO5*) in the Melonete-DB database (http://melonetdb.agbi.tsukuba.ac.jp/; Yano et al., 2018; 2020). The amino acid sequences of *CmACO*s were compared with those of agriculturally important species such as maize (ZmACOs), rice (OsACOs), tomato (SlACOs), cucmber (CsACOs), apple (MdACOs), and *Arabidopsis thaliana* (AtACOs) (Figure 1A). *CmACO1*, *CmACO3*, and *CmACO5* were categorized as Type I, *CmACO2* as Type II, and *CmACO4* as Type III. *CmACO1* was expressed only in the fruit postharvest, *CmACO3* in vegetative and reproductive organs, *CmACO4* in the roots, and *CmACO5* in vegetative and reproductive organs. Only *CmACO1* showed high expression in fruit, especially in the postharvest period, and was not expressed during the developmental stages (Figure 1B). Based on these findings, *CmACO1* was selected as the target gene for extending fruit shelf-life. To select the target site within *CmACO1*, essential residues for ACO activity were determined (Figure 1C) based on information regarding the ACC oxidase in apples (*MdACO1*) (Dilley et al., 2013), which were highly conserved in several agriculturally important species (Supplementary Figure S2; Houben and Van de Poel, 2019). We selected three target positions (Guide 1, Guide 3, and Guide 6) using CRISPR-P (http://crispr.hzau.edu.cn/CRISPR2/) in this region (Figure 1C).

### 3.2 Transformation of CRISPR/Cas9 system into melon genome via *Agrobacterium*-mediated transformation with tissue culture

**FIGURE 2:**
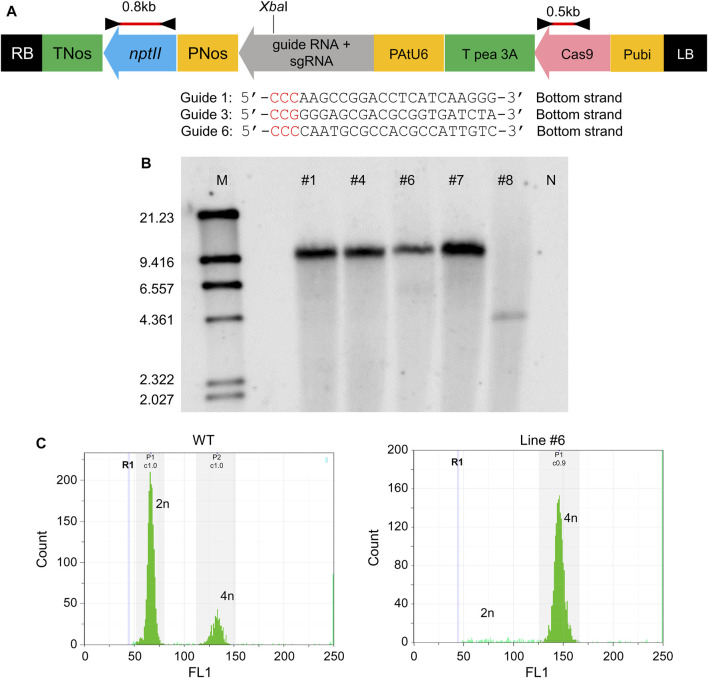
Establishment of melon lines created via CRISPR/Cas9 **(A)** Plasmid map of CRISPR/Cas9 system pDeCas9CmACO1. The black triangle shows the position of the primer set to amplify the 0.8 kb fragment (nptII region). The red bar indicates the probe for Southern blotting in **(B)**. **(B)** Southern blotting. M represents marker λ/HindIII. #1, #4, #6, #7, #8 are line numbers. N denotes genomic DNA from non-transgenic melon, the negative control. *nptII* region (0.8 kb) was used as probe (red bar in [A]). **(C)** Ploidy of transformant in **(B)**. W.T. was the control; line #1 was the same sample used in **(B)**. All transformants in **(B)** showed tetraploidy (data not shown).

Three kinds of genome editing vectors were transformed, each containing Guide 1, Guide 3, and Guide 6. The map of the transfer DNA (T-DNA) region of the binary vector is shown in Figure 2A. Regenerated shoots with roots appeared and acclimated to open-air conditions (Supplementary Figure S3). Genomic DNA was extracted from the shoots, and PCR confirmed the insertion of T-DNA with primer sets in Figure 2A. PCR test results were positive for five shoots, which came from the vector with Guide 6 only (data not shown). These lines were designated as #1, #4, #6, #7, and #8. The transformation efficiency was around 1% (regenerated shoots number per explants number, data not shown). The copy numbers in these five shoots were verified by Southern blotting (Figure 2B). All five shoots showed a single band, meaning that all of these shoots inserted a single copy of T-DNA in the region. The signals from four of the five shoots, Line #1, #4, #6, and #7, showed the same size, whereas the signal position of Line #8 differed. These findings indicated that all shoots were obtained with a single copy of the CRISPR-Cas9 gene and that Lines #1, #4, #6, and #7 were from the same shoot buds, but Line #8 was from an independent shoot. The ploidy of all five shoots was checked, and all transgenic T_0_ lines were tetraploid (Figure 2C).

### 3.3 Genotyping of the T_0_ generation

To detect the mutation, the target site on each transgenic line was cloned into a T-vector, and the resulting 54 sequence clones were checked by Sanger sequencing (Table 1). No large deletions or insertions were detected, and one-to-two base insertions, deletions, and substitutions were found 2–9 bp downstream of the PAM (NGG) sequence. The variations are deletion “a,” “t,” and “at,” insertion “a”, “t”, “at”, insertion “a,” and substitution “c” from “t” at the same time. Most of the sequenced clones (31/54; 57.4%) exhibited single or double nucleotide insertions, and 40.7% (22/54) showed single or double nucleotide deletions. Simultaneous insertions and substitutions were observed in 1.8% (1/54) of the clones, and no mutations were detected in the W.T. genotypes (0/54). The most commonly inserted nucleotide was “a” (25.9%), followed by “t” (0.7%). Lines #1 and #6 showed the same mutation pattern, whereas lines #4, #7, and #8 exhibited different mutation patterns. Each line contained three to four mutation patterns. Thus, the zygosity was predicted to be putatively chimeric in the T_0_ generation. Its nucleic acid sequence was translated to the corresponding amino acid sequence to verify the introduction of a stop codon at the region where amino acid residues important for enzyme activity were concentrated (Table 2). In all mutations, the stop codon introduced seven to eight amino acid residues downstream of the target sites, eliminating the back half of the regions where amino acid residues important for enzyme activity were concentrated. The mutation to defeat *CmACO1* was verified in the T_0_ generation. To produce seeds of the T_1_ generation, the five lines (Line #1, #4, #6, #7, and #8) were grown in the Special Netted-house. Although all five lines bore fruit, few seeds were produced, and most comprised only the seed coat (Data not shown). Moreover, Line #4 produced only a seed coat. Four lines (#1, #6, #7, and #8) were used to analyze the T_1_ generation.

**TABLE 1:** CRISPR/Cas9-induced mutations in target genes of the T_0_ generation.

Line	Genotype	Frequency	Nucleotide sequence
	W.T.		gac​gtg​c*ccc* **caa​tgc​gcc​acg​cca​ttg​tc**gtc​aac​ctc--
#1	d1(a)	4/11	gac​gtg​c*ccc* **ca-tgc​gcc​acg​cca​ttg​tc**gtc​aac​ctc--
	i1(a)	6/11	gac​gtg​c*ccc* **caa​atg​cgc​cac​gcc​att​gtc**gtc​aac​ctc--
	i1(t)	1/11	gac​gtg​c*ccc* **caa​ttg​cgc​cac​gcc​att​gtc**gtc​aac​ctc--
#4	d1(t)	4/12	gac​gtg​c*ccc* **caa-gcg​cca​cgc​cat​tgt​c**gtc​aac​ctc--
	d2 (a,t)	4/12	gac​gtg​c*ccc* **ca--gcg​cca​cgc​cat​tgt​c**gtc​aac​ctc--
	i1(a)	4/12	gac​gtg​c*ccc* **caa​ttg​cgc​cac​gcc​att​gtc**gtc​aac​ctc--
#6	d1(a)	3/10	gac​gtg​c*ccc* **ca-tgc​gcc​acg​cca​ttg​tc**gtc​aac​ctc--
	i1(a)	4/10	gac​gtg​c*ccc* **caa​atg​cgc​cac​gcc​att​gtc**gtc​aac​ctc--
	i1(t)	3/10	gac​gtg​c*ccc* **caa​ttg​cgc​cac​gcc​att​gtc**gtc​aac​ctc--
#7	d1(a)	3/9	gac​gtg​c*ccc* **ca-tgc​gcc​acg​cca​ttg​tc**gtc​aac​ctc--
	i1(a)	3/9	gac​gtg​c*ccc* **caa​atg​cgc​cac​gcc​att​gtc**gtc​aac​ctc--
	i1(t)	2/9	gac​gtg​c*ccc* **caa​ttg​cgc​cac​gcc​att​gtc**gtc​aac​ctc--
	i1(a), c→t	1/9	gac​gtg​c*ccc* **caa​atg​cgt​cac​gcc​att​gtc**gtc​aac​ctc--
#8	d1(a)	4/12	gac​gtg​c*ccc* **ca-tgc​gcc​acg​cca​ttg​tc**gtc​aac​ctc--
	i1(a)	6/12	gac​gtg​c*ccc* **caa​atg​cgc​cac​gcc​att​gtc**gtc​aac​ctc--
	i1(t)	1/12	gac​gtg​c*ccc* **caa​ttg​cgc​cac​gcc​att​gtc**gtc​aac​ctc--
	i2 (a,t)	1/12	gac​gtg​c*ccc* **caa​att​gcg​cca​cgc​cat​tgt​c**gtc​aac​ctc--

“d” and “i” means deletion and insertion, respectively. “→” indicates substitution. The number after “d” or “i” is the number of baspaires for deletion or insertion. The alphabed in the brackets shows the kinds of the nucleotide for deletion or insertion. “W.T.” is the wilde type genotype. Bold and characters with under line represent the target and mutation sequences, respectively. Italic means 3′-NGG sequence.

**TABLE 2:** Amino acid sequences of target sites (T_0_ generation).

Line	Genotype	Amino acid sequence
	W.T.	RA ***H*** T ***D***-24aa-IDV**PPMRHAIV**VNLGDQLEVITNGRYKSVM ***H*** R-94aa-A*
#1	d1(a)	RA ***H*** T ***D*** -24aa-IDVPPCATPLSSTSGTNLR*
	i1(a)	RA ***H*** T ***D*** -24aa-IDVPPNAPRHCRQPRGPT*
	i1(t)	RA ***H*** T ***D*** -24aa-IDVPPIAPRHCRQPRGPT*
#4	d1(t)	RA ***H*** T ***D*** -24aa-IDVPPSATPLSSTSGTNLR*
	d2 (a,t)	RA ***H*** T ***D*** -24aa-IDVPPNCATPLSSTGTNLR*
	i1(a)	RA ***H*** T ***D*** -24aa-IDVPPNAPRHCRQPRGPT*
#6	d1(a)	RA ***H*** T ***D*** -24aa-IDVPPCATPLSSTSGTNLR*
	i1(a)	RA ***H*** T ***D*** -24aa-IDVPPNAPRHCRQPRGPT*
	i1(t)	RA ***H*** T ***D*** -24aa-IDVPPIAPRHCRQPRGPT*
#7	d1(a)	RA ***H*** T ***D*** -24aa-IDVPPCATPLSSTSGTNLR*
	i1(a)	RA ***H*** T ***D*** -24aa-IDVPPNAPRHCRQPRGPT*
	i1(t)	RA ***H*** T ***D*** -24aa-IDVPPIAPRHCRQPRGPT*
	i1(a), c→t	RA ***H*** T ***D*** -24aa-IDVPPIASRHCRQPRGPT*
#8	d1(a)	RA ***H*** T ***D*** -24aa-IDVPPCATPLSSTSGTNLR*
	i1(a)	RA ***H*** T ***D*** -24aa-IDVPPNAPRHCRQPRGPT*
	i1(t)	RA ***H*** T ***D*** -24aa-IDVPPIAPRHCRQPRGPT*
	i2 (a,t)	RA ***H*** T ***D*** -24aa-IDVPPAPRHCRQPRGPT*

Italic characters represent essential amino acid residues for ACC oxidase activity. The underline means the amino acid sequence altered by CRISPR/Cas9. The bold is a target site.

### 3.4 Germination ratio and genotyping of the T_1_ generation

To check the germination ratio in the T_1_ generation, 15 seeds were sown for each line. Only two to three seeds germinated, with germination ratios of 13.3%–20% (Supplementary Table S1). To verify the genotype of the T_1_ generation, the genomic DNA was extracted from the cotyledon of two or three seedlings in each line. PCR amplified the target site, and the fragment was cloned into the pGEM-T vector (Promega); 102 clones were analyzed by Sanger sequencing (Table 3). In the third seedling of the T_1_ generation of Line #1 (#1–3) and the fifth seedling of the T_1_ generation of Line #7 (#7–5), T-DNA insertion was detected, and a new mutation pattern was identified (insertions “aa” and “at” in Line #1–3; substitution “a” to “t” and deletion of five nucleotides in Line #7–5). In Line #6-2, the W.T. genotype was identified with the deletion “a” and the insertion “a”, as seen in the T_0_ generation. The other lines showed the same mutation as that in the T_0_ generation. Line #7-1 harbored one mutation pattern, and #6-4 contained two. The zygosity of these lines was predicted as homo- and bi-allelic, respectively. The other lines showed more than three mutation patterns, resulting in chimeric zygosity. The nucleotide sequences of these clones were translated into amino acid sequences (Table 4). Except for the “a” to “t” substitution in Lines #7-5, all mutations were associated with a stop codon downstream of five to nine amino acid residues from the target sites. These mutations indicated that the essential structure for ACO activity was removed. The substitution of nucleotide “a” to “t” changed an amino acid residue at position 209 from “M" to “K" (M209K). PCR also confirmed T-DNA insertion with primer sets to amplify the *nptII* and *Cas9* regions (Table 3, primer position is indicated in Figure 2A). Line #1–3, #1–4, #6–2, #6–4, #7–1, #8–2, #8-3, and #8-6 were PCR negative for both of *nptII* and *Cas9*, which means that these lines did not contain the T-DNA region in their genome. On the other hand, the *nptII* and *Cas9* region was detected in Lines #7–5; only this line contained the T-DNA region in its genome.

**TABLE 3:** CRISPR/Cas9-induced mutations in target genes of the T_1_ generation.

Line	Genotype	Frequency	Nucleotide sequence	T-DNA
	WT		gac​gtg​c*ccc* **caa​tgc​gcc​acg​cca​ttg​tc**gtc​aac​ctc--	
#1–3	d1(a)	5/14	gac​gtg​c*ccc* **ca-tgc​gcc​acg​cca​ttg​tc**gtc​aac​ctc--	Positive
	i2 (at)	2/14	gac​gtg​c*ccc* **caa​att​gcg​cca​cgc​cat​tgt​c**gtc​aac​ctc-	
	i2 (aa)	1/14	gac​gtg​c*ccc* **caa​aat​gcg​cca​cgc​cat​tgt​c**gtc​aac​ctc-	
	i1(a)	3/14	gac​gtg​c*ccc* **caa​atg​cgc​cac​gcc​att​gtc**gtc​aac​ctc--	
	i1(t)	3/14	gac​gtg​c*ccc* **caa​ttg​cgc​cac​gcc​att​gtc**gtc​aac​ctc--	
#1–4	d1(a)	1/8	gac​gtg​c*ccc* **ca-tgc​gcc​acg​cca​ttg​tc**gtc​aac​ctc--	Negative
	i1(a)	4/8	gac​gtg​c*ccc* **caa​atg​cgc​cac​gcc​att​gtc**gtc​aac​ctc--	
	i1(t)	3/8	gac​gtg​c*ccc* **caa​ttg​cgc​cac​gcc​att​gtc**gtc​aac​ctc--	
#6–2	WT	1/16	gac​gtg​c*ccc* **caa​tgc​gcc​acg​cca​ttg​tc**gtc​aac​ctc--	Negative
	d1(a)	10/16	gac​gtg​c*ccc* **ca-tgc​gcc​acg​cca​ttg​tc**gtc​aac​ctc--	
	i1(a)	5/16	gac​gtg​c*ccc* **caa​atg​cgc​cac​gcc​att​gtc**gtc​aac​ctc--	
#6–4	i1(a)	7/13	gac​gtg​c*ccc* **caa​atg​cgc​cac​gcc​att​gtc**gtc​aac​ctc--	Negative
	i1(t)	6/13	gac​gtg​c*ccc* **caa​ttg​cgc​cac​gcc​att​gtc**gtc​aac​ctc--	
#7–1	i1(a)	14/14	gac​gtg​c*ccc* **caa​atg​cgc​cac​gcc​att​gtc**gtc​aac​ctc--	Negative
#7–5	a→t	1/14	gac​gtg​c*ccc* **caa​agc​gcc​acg​cca​ttg​tc**gtc​aac​ctc--	Positive
	a→t, d5	3/14	gac​gtg​c*ccc* **caa​a-----acg​cca​ttg​tc**gtc​aac​ctc--	
	i1(t)	10/14	gac​gtg​c*ccc* **caa​ttg​cgc​cac​gcc​att​gtc**gtc​aac​ctc--	
#8–2	d1(a)	3/6	gac​gtg​c*ccc* **ca-tgc​gcc​acg​cca​ttg​tc**gtc​aac​ctc--	Negative
	i1(a)	1/6	gac​gtg​c*ccc* **caa​atg​cgc​cac​gcc​att​gtc**gtc​aac​ctc--	
	i1(t)	2/6	gac​gtg​c*ccc* **caa​ttg​cgc​cac​gcc​att​gtc**gtc​aac​ctc--	
#8–3	d1(a)	1/11	gac​gtg​c*ccc* **ca-tgc​gcc​acg​cca​ttg​tc**gtc​aac​ctc--	Negative
	i1(a)	5/11	gac​gtg​c*ccc* **caa​atg​cgc​cac​gcc​att​gtc**gtc​aac​ctc--	
	i1(t)	5/11	gac​gtg​c*ccc* **caa​ttg​cgc​cac​gcc​att​gtc**gtc​aac​ctc--	
#8–6	d1(a)	2/6	gac​gtg​c*ccc* **ca-tgc​gcc​acg​cca​ttg​tc**gtc​aac​ctc--	Negative
	i1(a)	1/6	gac​gtg​c*ccc* **caa​atg​cgc​cac​gcc​att​gtc**gtc​aac​ctc--	
	i1(t)	3/6	gac​gtg​c*ccc* **caa​ttg​cgc​cac​gcc​att​gtc**gtc​aac​ctc--	

Bold and characters with under line represent the target and mutation sequences, respectively. Italic means 3′-NGG sequence.

**TABLE 4:** Amino acid sequences of target sites (T_1_ generation).

Line	Geenotype	Aminoacid sequence
	WT	RA ***H*** T ***D***-24aa-IDV**PPMRHAIV**VNLGDQLEVITNGRYKSVM ***H*** R-94aa-A*
#1–3	d1(a)	RA ***H*** T ***D***-24aa-IDVPPCATPLSSTSGTNLR*
	i2 (at)	RA ***H*** T ***D***-24aa-IDVPPNCATPLSSTSGTNLR*
	i2 (aa)	RA ***H*** T ***D***-24aa-IDVPPKCATPLSSTSGTNLR*
	i1(a)	RA ***H*** T ***D***-24aa-IDVPPNAPRHCRQPRGPT*
	i1(t)	RA ***H*** T ***D***-24aa-IDVPPIAPRHCRQPRGPT*
#1–4	d1(a)	RA ***H*** T ***D***-24aa-IDVPPCATPLSSTSGTNLR*
	i1(a)	RA ***H*** T ***D***-24aa-IDVPPNAPRHCRQPRGPT*
	i1(t)	RA ***H*** T ***D***-24aa-IDVPPIAPRHCRQPRGPT*
#6–2	WT	RA ***H*** T ***D***-24aa-IDVPPMRHAIVVNLGDQLEVITNGRYKSVM ***H*** R-94aa-A*
	d1(a)	RA ***H*** T ***D***-24aa-IDVPPCATPLSSTSGTNLR*
	i1(a)	RA ***H*** T ***D***-24aa-IDVPPNAPRHCRQPRGPT*
#6–4	i1(a)	RA ***H*** T ***D***-24aa-IDVPPNAPRHCRQPRGPT*
	i1(t)	RA ***H*** T ***D***-24aa-IDVPPIAPRHCRQPRGPT*
#7–1	i1(a)	RA ***H*** T ***D***-24aa-IDVPPNAPRHCRQPRGPT*
#7–5	a→t	RA ***H*** T ***D***-24aa-IDVPPKRHAIVVNLGDQLEVITNGRYKSVM ***H*** R-94aa-A*ATA*
	a→t, d5	RA ***H*** T ***D***-24aa-IDVPPKRHCRQPRGPT*
	i1(t)	RA ***H*** T ***D***-24aa-IDVPPIAPRHCRQPRGPT*
#8–2	d1(a)	RA ***H*** T ***D***-24aa-IDVPPCATPLSSTSGTNLR*
	i1(a)	RA ***H*** T ***D***-24aa-IDVPPNAPRHCRQPRGPT*
	i1(t)	RA ***H*** T ***D***-24aa-IDVPPIAPRHCRQPRGPT*
#8–3	d1(a)	RA ***H*** T ***D***-24aa-IDVPPCATPLSSTSGTNLR*
	i1(a)	RA ***H*** T ***D***-24aa-IDVPPNAPRHCRQPRGPT*
	i1(t)	RA ***H*** T ***D***-24aa-IDVPPIAPRHCRQPRGPT*
#8–6	d1(a)	RA ***H*** T ***D***-24aa-IDVPPCATPLSSTSGTNLR*
	i1(a)	RA ***H*** T ***D***-24aa-IDVPPNAPRHCRQPRGPT*
	i1(t)	RA ***H*** T ***D***-24aa-IDVPPIAPRHCRQPRGPT*

Italic characters represent essential amino acid residues for ACC oxidase activity. The underline means the amino acid sequence altered by CRISPR/Cas9. The bold is a target site.

### 3.5 Characteristics of the T_1_ generation

**FIGURE 3:**
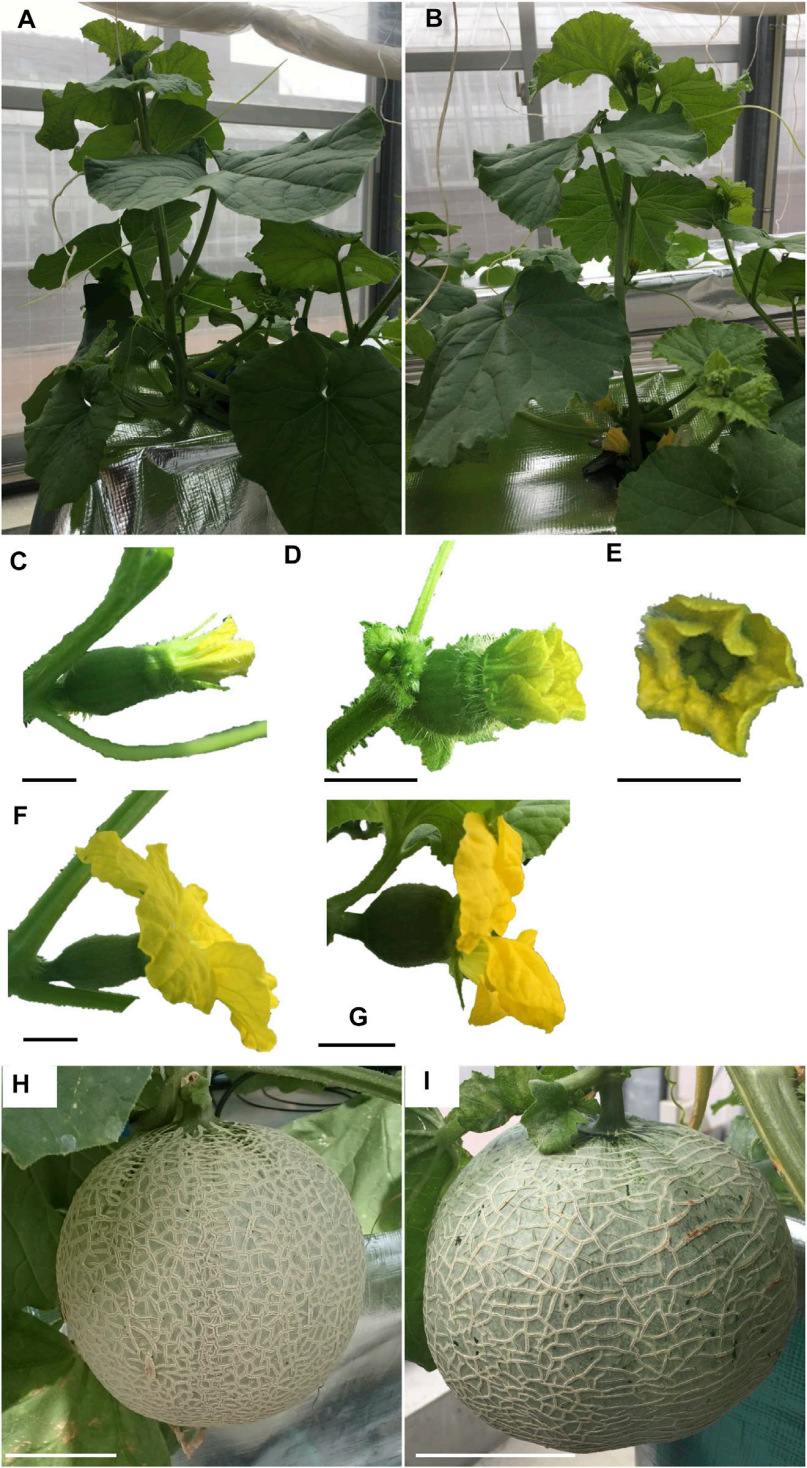
Growth of T_1_ melons transformed with CRISPR/Cas9. **(A, B)** show plant posture. **(C–E)** show buds 1 day before flowering. **(F, G)** show flowers. **(H)** and **(I)** show fruits 24d after pollination. **(A)**, **(C)**, **(F)**, and **(H)** show diploid non-transformant plants with WT *CmACO1*. **(B)**, **(D)**, **(E)** and **(I)** are tetraploid, CRISPR/Cas9-modified plants with a mutation of *CmACO1*. Bars in **(C–G)** show 1 cm. Bars in **(H, I)** are 10 cm. **(A)**, **(C)**, **(F)** and **(H)** are W.T. plants. **(B)**, **(D)**, **(E)**, **(G)**, and **(I)** were Lines #6–4.

**FIGURE 4:**
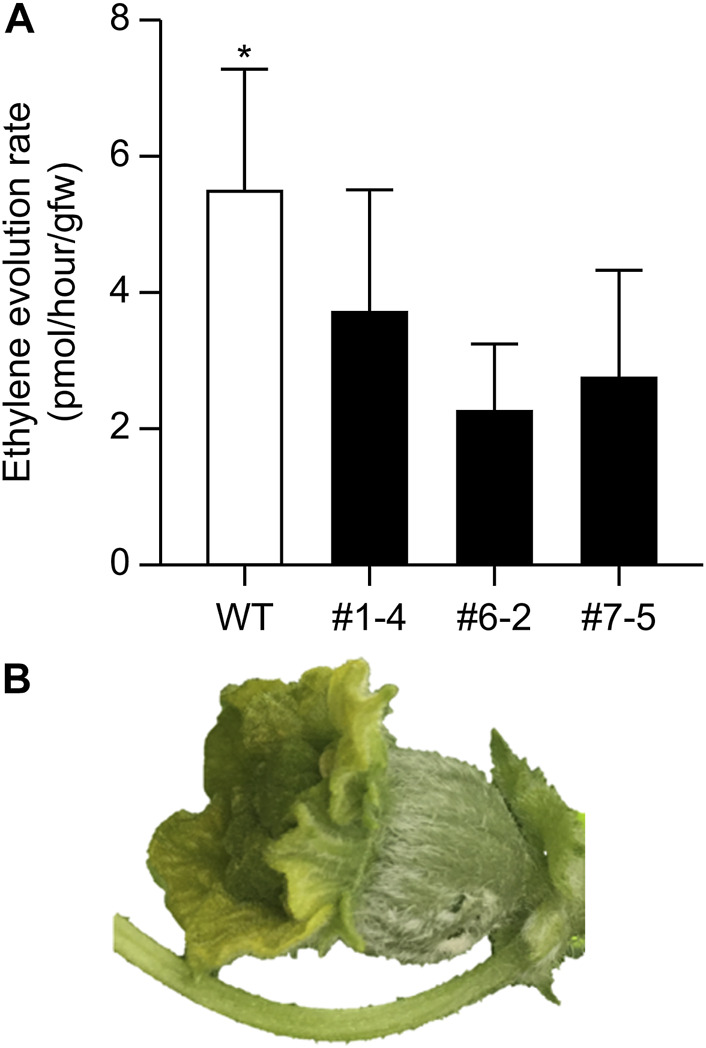
Ethylene evolution from apical shoots and induction of female flowers via ethylene treatment. **(A)** Detection of ethylene from apical shoots. Bars indicate standard deviation (*n* = 3); asterisk indicates significant differences by Student’s t-test (*p* < 0.05). **(B)** Female buds induced by 1% ethephon treatment.

The nine lines listed in Table 4 of the T_1_ generation were grown in the Special Netted-House. Line #6-4 grew normally, showing no notable (Figures 3A, B). However, differences were observed in flower morphology between #6-4 and W.T. lines, particularly regarding the ovary, petals, and stigma (Figures 3C–G). The ovaries of Lines #6-4 were more spherical than those of the W.T. (Figures 3C–G). The stigma was covered with petals on the bud 1 day before flowering in the W.T. line, but in lines #6-4, the stigma was uncovered (Figure 3E). The flowers were self-pollinated, and the fruit set was observed in the W.T. and Line #6–4 (Figures 3H, I). Line #6-4 yielded smaller, flatter than the W.T. Lines #1–4, #6-2, and #7-5 developed only male flowers (androecious phenotype). Ethylene production is involved in the induction of female flowers; thus, we hypothesized that the apical shoot and floral meristem failed to produce ethylene because ACC oxidase activity was blocked, inhibiting female flowers’ formation. To investigate this hypothesis, ACC oxidase activity was measured in the apical shoots of Lines #1–4, #6-2, and #7–5. ACC oxidase activity was detected as ethylene evolution after adding ACC, an immediate precursor of ethylene. Ethylene production in all three lines was approximately half that of the W.T (Figure 4A). To investigate whether ethylene shortage caused this phenomenon, shoot apexes were treated with ethephon. Several weeks after the treatment, female flowers with abnormal shape relative to W.T. flowers appeared in Lines #1–4, #6-2, and #7–5 (Figure 4B). These flowers had short petals and an open stigma on the flower bud; they failed to set fruit. Mutation Lines #1–3, #7–1, #8–2, #8-3, and #8-6 exhibited gummy stem blight on the base of the stalk and died (Data not shown). Only Lines #6-4 succussed the production seed of T_2_ generation; the total seeds number was less than 30 seeds per fruit, which was quite low. The shape of the seeds was broader and thicker than W.T. This line was used for the evaluation of fruit shelf life.

### 3.6 Extension of shelf-life in T_2_ fruits

**FIGURE 5:**
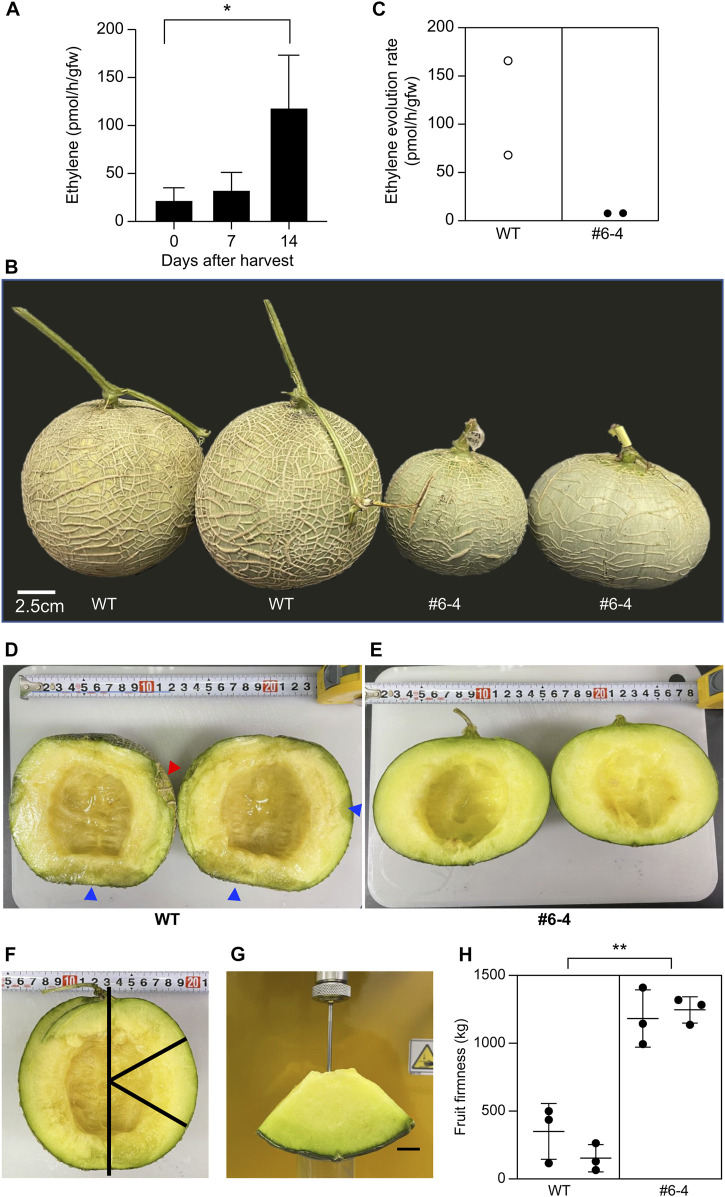
Evaluation of shelf-life in melon with modified *CmACO1* **(A)** Ethylene evolution rate of W.T. melon. Bars indicate standard deviation; asterisk shows significant differences by one-way ANOVA (*p* < 0.05) (*n* = 4). **(B)** Melon fruits 14 d after harvest (stored at 25°C). W.T., wild type; #6-4, mutation line. **(C)** Ethylene evolution rate from melon fruit 14 d after harvest. **(D, E)** are the longitudinal section of fruit. **(F, G)** are the measurement of fruit firmness. **(H)** Fruit firmness. Bars indicate standard deviation; asterisk shows significant differences by one-way ANOVA (*p* < 0.05) (*n* = 3).

As a preliminary test to decide the timing of the measurement of ethylene evolution, the amount of ethylene produced in wild-type fruit was measured 0, 7, and 14 days after harvest. The ethylene evolution rates from W.T. melon fruit on 0, 7, and 14 days after harvest were 22.23 ± 12.78, 32.82 ± 17.93, and 118.6 ± 54.70 pmol/h/gfw, respectively (Figure 5A). Fourteen days after harvest, ethylene evolution was four times higher than that measured 7 days after harvest. Therefore, 14 was chosen as the best time for this experiment’s measurement of ethylene evolution and storage period. Plants were sown and cultivated in the netted greenhouse to evaluate the shelf-life of T_2_ generation fruit from the mutant Line #6–4. Each plant exhibited the genotype insertion “a” and “t”, confirming biallelic zygosity; PCR did not detect T-DNA. The shape and size of the ovary and flower in the T_2_ generation are almost the same as in T_1_ generation (Data not shown). To evaluate shelf-life, pericarp color, ethylene evolution, and fruit flesh were analyzed 14 days after harvest (Figure 5B). The color of the pericarp changed from green to yellow in W.T. fruits but remained green in Lines #6-4 fruits (Figure 5B). Fourteen days after harvest, the ethylene evolution rate in W.T. fruits was 0.68 and 1.67 pmol/h/gfw, whereas that in Line #6-4 fruits was 0.079 and 0.078 pmol/h/gfw (Figure 5C). To compare fruit quality between W.T. and mutant Line #6-4, the fruits were cut longitudinally (Figures 5D, E). The pericarp was quite soft in W.T. fruits, resulting in collapse upon cutting (red arrowhead in Figure 5D). The cross-sectional analysis of W.T. fruit revealed a wet, mushy texture (blue arrowhead in Figure 5D) without a fermented odor, as is representative of early fermentation. In contrast, fruits of Line #6-4 did not show pericarp collapse or mushy texture (Figure 5E). Evaluation of fruit firmness showed that the maximum strength of W.T. fruit was one-third to one-tenth that of Line #6-4 fruit, indicating greater softness (Figures 5F–H).

## 4 Discussion

In this study, we attempted to introduce a long shelf-life trait in melon fruit by modifying *CmACO1* via the CRISPR/Cas9 system. Although the melon genome contains five ACC oxidase genes, only *CmACO1* was expressed in postharvest fruit (Figure 1). Moreover, compared to W.T., the ethylene level was completely reduced in Lines #6-4, which contained a *CmACO1* mutation (Figure 5C). This suggests that other ACO homologs (CmACO2, CmACO3, CmACO4, and CmACO5) did not express in the fruit postharvest. ACC oxidase activity is typically evaluated by measuring ethylene evolution (Khan and Singh, 2007), which indicates ACC oxidase activity. Compared to W.T. fruit, ethylene production was quite a low level in the mutant Line #6-4 postharvest, under the same condition, indicating that the induced mutation in *CmACO1* via CRISPR/Cas9 suppressed *CmACO1* activity in melon. The mutation introduced by CRIPSR/Cas9 is precise and quite stable beyond the generation. Previous studies have demonstrated that progeny can inherit mutations induced by CRISPR/Cas9 in melon and tomato (Ito et al., 2015; Lee et al., 2018; Giordano et al., 2022). Moreover, the inhibition of *CmACO1* by EMS treatment prevents ethylene production in fruit (Dahmani-Mardas et al., 2010). In our study, the mutation of *CmACO1* induced by CRISPR/Cas9 was inherited by the T_1_ and T_2_ generation. In the T_2_ generation, Line #6-4 only had bi-allaric insertions “a” and deletions “a”; thus, it is reasonable to assume that the mutation can be detected in the T_3_ generation of Line #6-4.

Five regenerated transformants with CRISPR/Cas9 had the mutation in this experiment; the rate of mutation induction was 100%. In a recent study that utilized a CRISPR/Cas9 system in melon, 40%–46% of transgenic lines with CRISPR/Cas9 had a mutation in the target gene (Giordano et al., 2022). Our results, along with the findings of previous reports, indicate that the CRISPR/Cas9 system is advantageous for introducing site-specific mutations with high efficiency, regardless of the target sights. Introducing CRISPR/Cas9 into the plant with high efficiency is essential to use this technique. In our experiment, the transformation frequency was low (0%–1%) and differed depending on the transformation vectors. Therefore, modifying the introduction methodology for increasing the transformation frequency is required to generalise this technology as a breeding technique. Four of the five (Lines #1, #4, #6, and #7) showed the same signal on the Southern hybridization analysis (Figure 2B), which meant that the insertion position of CRISPR/Cas9 in the genome was the same and that Lines #1, #4, #6, and #7 are clones. However, the mutation pattern differed; if the CRISPR/Cas9 worked only after insertion, the mutation pattern would be the same among clones. The differences in mutation patterns among the clones indicate that CRISPR/Cas9 may work after insertion into the plant genome. This may continue until later in the regeneration process during transformation in the T_0_ generation.

The acidity and total soluble sugars are important factors in determining fruit quality during postharvest. A previous study found that suppressing the *CmACO1* gene with an antisense system did not affect total soluble sugar content (Ayub et al., 1996). Moreover, there were no significant differences in soluble sugars between wild type and mutant lines of *CmACO1* with EMS treatment (Dahmani-Mardas et al., 2010). Therefore, we expect that our mutation Line #6-4 will not show significant differences in soluble sugar content compared to the W.T. line. The cuticle is another vital factor in determining fruit quality during postharvest. The composition and architecture of the cuticle affect fruit softening in peppers, tomatoes, and blueberries (Bargel and Neinhuis, 2004; Maalekuu et al., 2005; Kosma et al., 2010; Moggia et al., 2016; Moggia et al., 2017). Previous studies have shown that ethylene is involved in some cuticular compounds in apples and oranges under cold storage conditions (Li et al., 2017; Romero and Lafuente, 2022). Exogenous ethylene alters epicuticular layer morphology and increases cell membrane permeability. Treatment with 1-MCP, an ethylene signal transduction inhibitor, delays wax constituents’ development (Curry, 2008). Based on these results, we expected that the component of cuticular properties would be altered in the mutant that reduces ethylene production via defeated *CmACO1*. However, the firmness of the fruit was much higher in the *CmACO1* mutation line (Figure 5C). From these results, we can infer that the cuticular components are not critical in determining the firmness of melon fruit. Ethylene controls the expression of genes related to fruit softening, such as pectin methyl esterase (PME), polygalacturonase (P.G.s), Endo-1,4-beta glucanase (EGase), and beta-galactosidase (beta-GAL), during postharvest (Nishiyama et al., 2007; Zhang et al., 2022). Our results indicate that the CRISPR/Cas9 system effectively suppressed *CmACO1*, leading to considerably lower ethylene levels than W.T (Figure 5C). and substantially firmer fresh fruit than W.T (Figure 5H). at 14 days after harvest. Our results, coupled with those of previous studies, suggest that inhibition of ethylene evolution in melon fruit does not trigger the expression of genes related to fruit softening, ultimately preserving the firmness of the fruit. Cell wall constituents, such as pectin, polygalacturonate, and 1,4-beta-glucans, are expected to play a more critical role in maintaining firmness than the outer epidermal cell wall component melon fruit.

Fruit size is another essential agronomic trait. A recent study on cucumbers showed that ethylene affects fruit size (Xin et al., 2019). However, in a study on melons, despite the suppression of the *CmACO1* gene expression via an antisense system and defeat of *CmACO1* via EMS mutagen reducing ethylene production in melon fruit, no significant differences in fruit size were reported (Ayub et al., 1996; Dahmani-Mardas et al., 2010). Moreover, the knockout of *CmNAC-NOR* via CRISPR/Cas9 suppressed ethylene evolution but did not show differences in fruit size (Liu et al., 2022). Notably, *CmACO1* is only expressed after the postharvest stage, not during fruit development (Figure 1B). Therefore, defeated *CmACO1* would be restrictive for only storage and not for other locations, such as fruit growth stages, including cell expansion and cell division. In general, the tetraploid plants exhibited larger fruit size than the diploid plants, but in melon, the relationships between the polyploidy and the fruit size vary and depend on the cultivars (Batra, 1952; Ezura et al., 1992a; Ezura et al., 1992b; Fassuliotis and Nelson, 1992; Zhang et al., 2010). For instance, in the derivative of ‘Earl’s favourite,’ the same cultivar used in our experiment, tetraploid fruit showed a smaller size and flattened shape (Ezura et al., 1992a; Ezura et al., 1992b). Based on these results, we can conclude that in this experiment, fruit size and shape in tetraploid with defeated *CmACO1* of ‘Earl’s favourite’ are associated with ploidy rather than the reduction of ethylene level.

The final fruit size is controlled by cell division, and cell expansion contributes considerably to it (van der Knaap and Østergaard, 2018). The increasing cell division and enlargement of cell size result in increased fruit size (Gillaspy et al., 1993; Xiao et al., 2009). However, the genome-edited Line #6-4 with tetraploid exhibited a smaller fruit size. Because fruit size depends on cell division and expansion, it may be presumed that inhibiting cell division and expansion in the pericarp would reduce fruit size substantially firmer fresh fruit. However, the reduction in fruit size would decrease the market value of gene-edited fruits, which is a concern that must be resolved. The new melon line with a long shelf-life (Line #6–4) in this study showed a smaller size due to tetraploidy. Previous studies showed tetraploids reduce fruit size in a melon ‘Earl’s favourite’ derivatives (Ezura et al., 1992a; Ezura et al., 1992b). Therefore, changing the ploidy would help overcome the issue of smaller fruit sizes. Crossbreeding diploid and tetraploid melon cultivars could enable the production of triploid melons (Ezura et al., 1993). Based on this finding, triploids with the valuable trait of long shelf-life would be produced by crossing tetraploid “Earl’s favourite Harukei-3” melons containing mutant *CmACO1* with diploids. Triploid melons have spherical fruit like diploid plants, with almost the same Brix value, and contain only empty seeds, which prevents seed outflow (Ezura et al., 1993). Therefore, crossbreeding diploid and tetraploid would be a useful method to overcome fruit size decreases. Another methodology is utilizing the “doubled haploid production in melon (Hooghvorst et al., 2020),” which produces doubled haploid lines from diploid melon. This method uses devitalized pollen via irradiation (Hooghvorst et al., 2020). Pollination of a female flower stigma with devitalized pollen stimulates an *in situ* parthenogenetic response when the pollen tube reaches the egg cell. The parthenogenetic haploid embryo is then developed, extracted, and cultured *in vitro*. The germinated embryo regenerates into a fully-developed plantlet that needs to undergo chromosome duplication for D.H. seed recovery. A diploid plant will be produced if this method, without the chromosome duplication step, is adopted for the tetraploid plant. Based on this knowledge, the tetraploid “Earl’s favourite Harukei-3” melons containing mutated *CmACO1* with long shelf-life can be utilized for melon breeding.

This study proposed a breeding method to extend shelf-life of “Earl’s favourite Harukei-3”, which is a parental line for breeding exclusive Japanese melon cultivars and representing essential export crops, using genome editing technology. Exporting fruits and vegetables requires close control of temperature, humidity, oxygen, and carbon dioxide to maintain fruit quality and freshness, leading to high energy usage and cost (Lamberty and Kreyenschmidt, 2022). Creating parent lines with a long shelf-life can reduce the energy and costs required for export quality control. Because the extending of shelf life is also related to the reduction of food loss and wast, this methodology, which we introduced in this study, would provide the contribution to increase the sustainability of the global food system, not only to reduce the cost for export. It follows from this that the genome editing technology has the possibility for the reduction of “food loss and waste” and to contribute for food security.

## Data Availability

The raw data supporting the conclusion of this article will be made available by the authors, without undue reservation.

## Supplementary material

Supplementary Figure S1 Measurement of ethylene from apical shoot of melon.

Supplementary Figure S2
**(A)** Comparison of amino acid sequence of MdACO1 and CmACO1. **(B–E)** Comparison of amino acid sequence among agricultural important crops and vegetables. Bule, black, red, and green dots were essential for ACO activity, binding site for ascorbate, and bicarbonate binding sites, respectively. **(F)** Comparison of amino acid sequence among ACOs in *C. melo* cv. Harukei-No.3.

Supplementary Figure S3 Transformant with CRISPR/Cas9 in T_0_ generation. **(A)** Regenerated shoots. **(B)** Acclimatization.
