# Supplementary figures and images for: Targeted modification of CmACO1 by CRISPR/Cas9 extends the shelf-life of Cucumis melo var. reticulatus melon
Source: Front Genome Ed. 2023 May 25;5:1176125. doi: 10.3389/fgeed.2023.1176125 (PMC10249633; doi:10.3389/fgeed.2023.1176125)

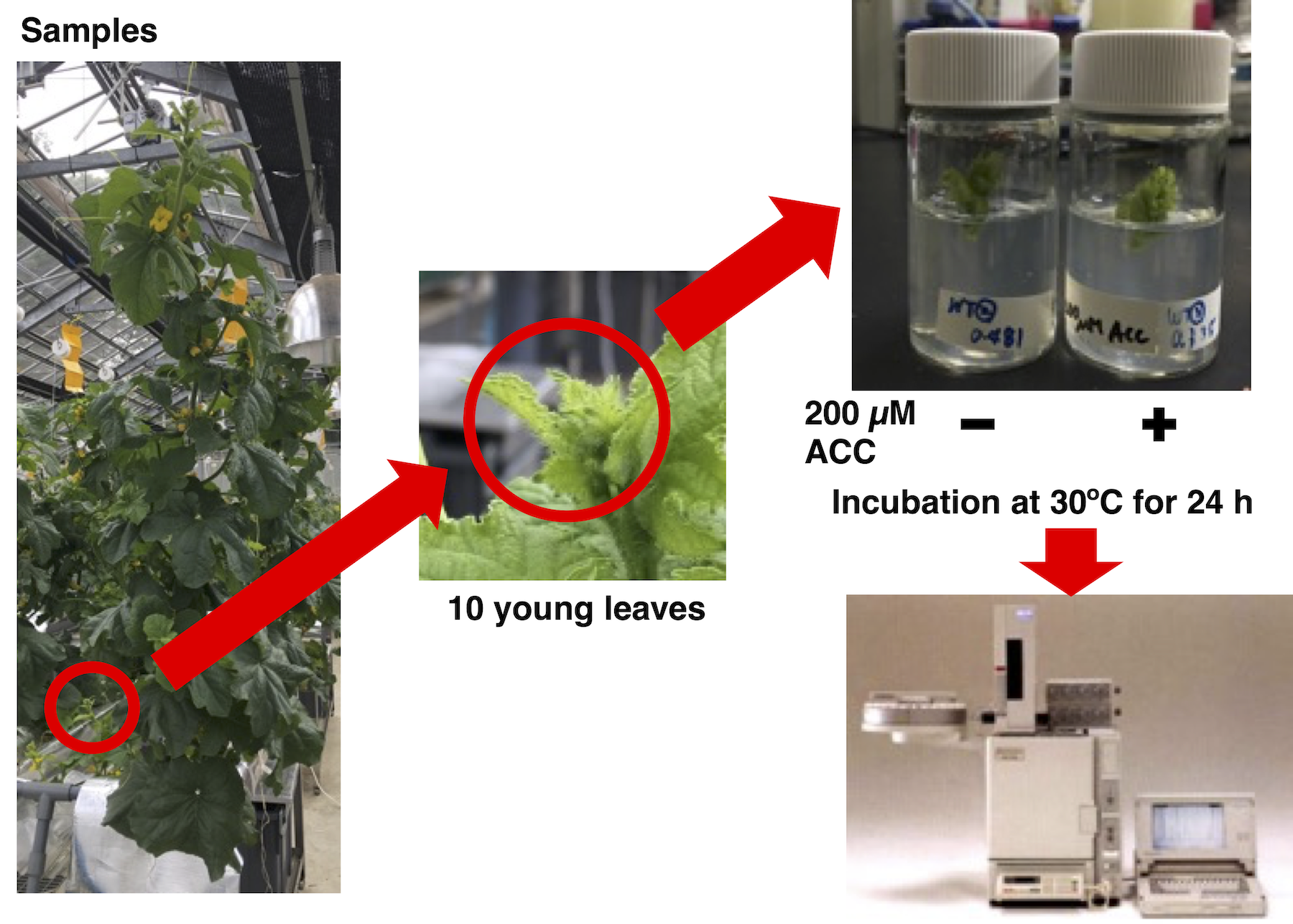

Supplement: Supplementary file 2 [file Image1.tiff]

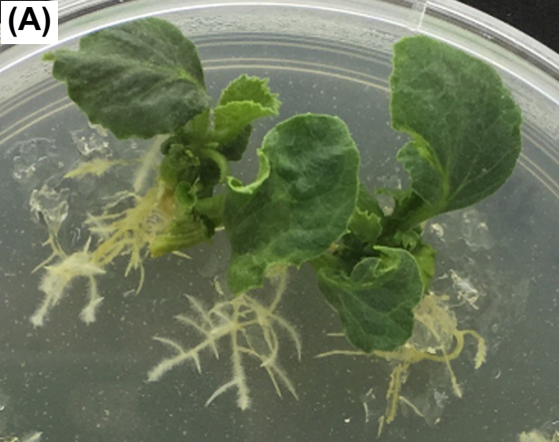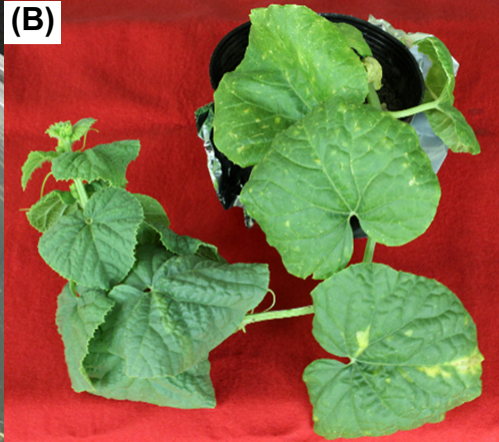

Supplement: Supplementary file 3 [file DataSheet4.PDF]

(E)

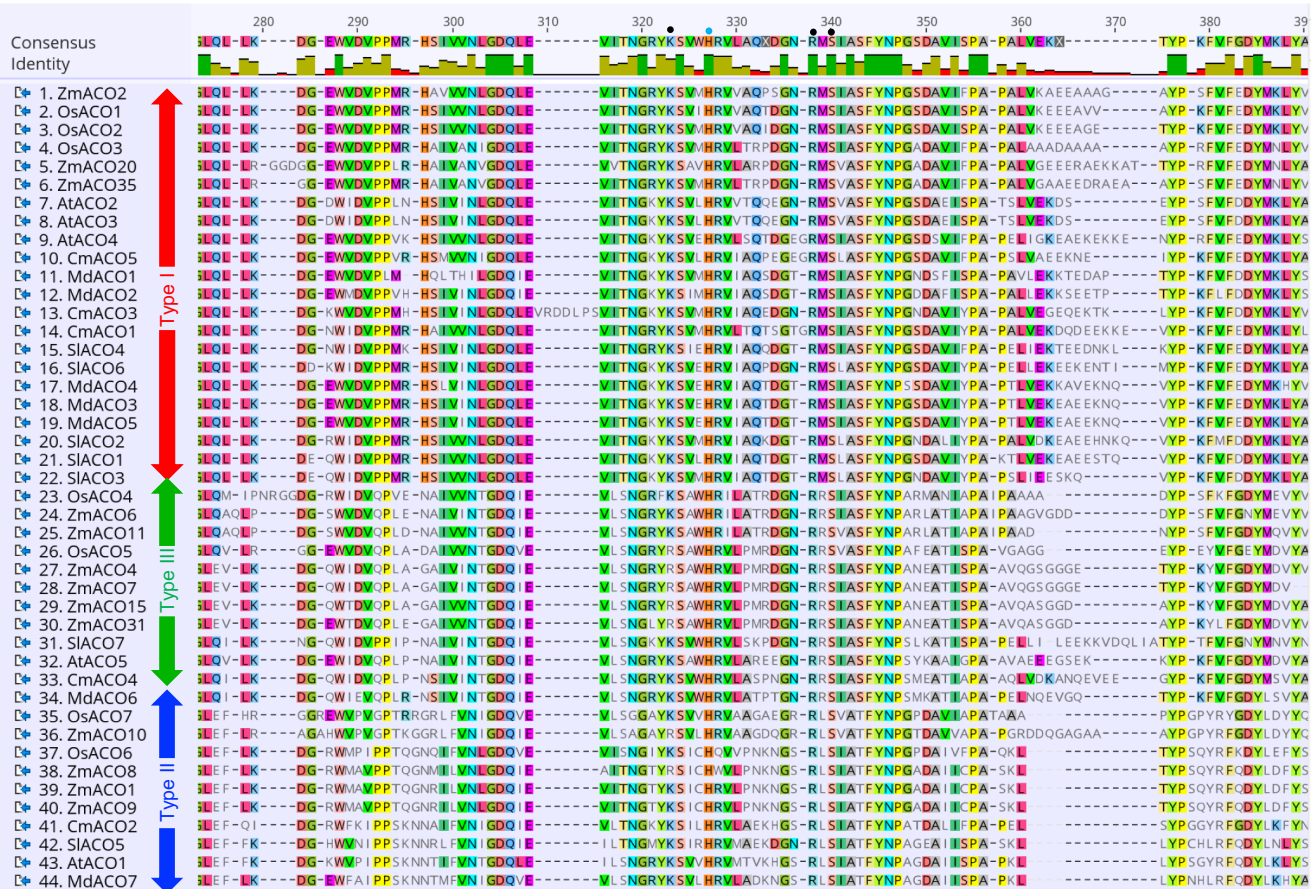

(F)

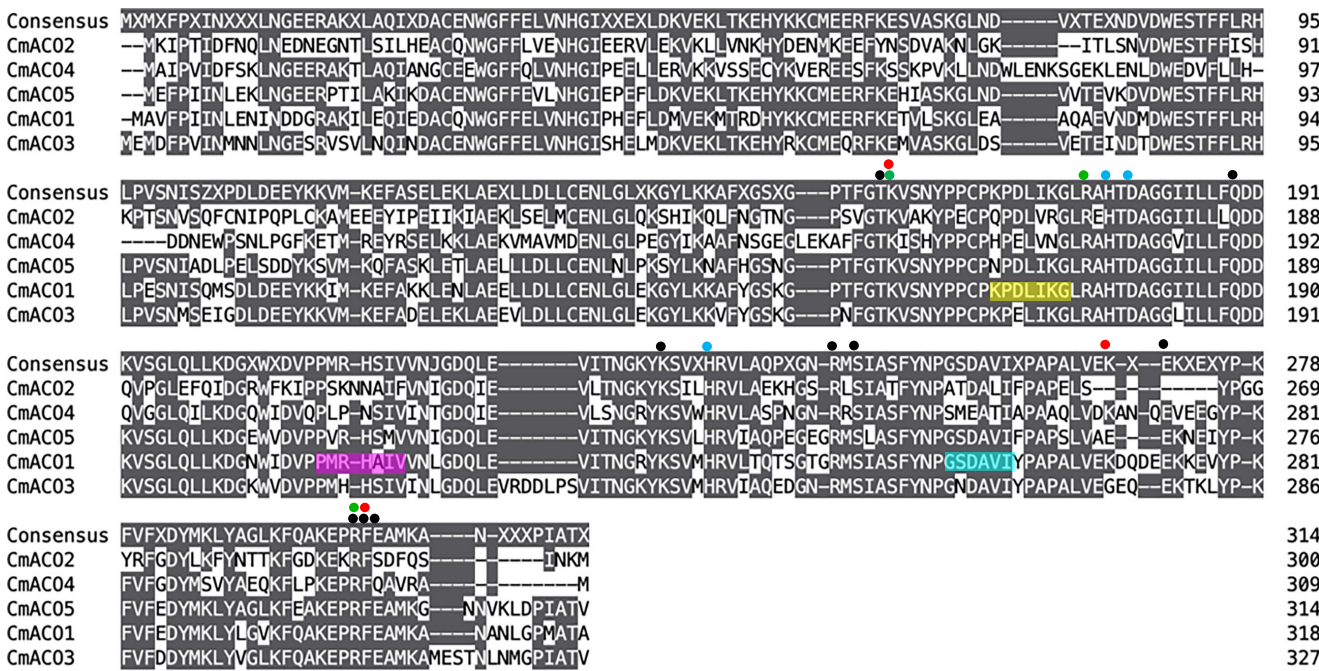

Supplement: Supplementary file 4 [file DataSheet3.PDF]

**Samples**

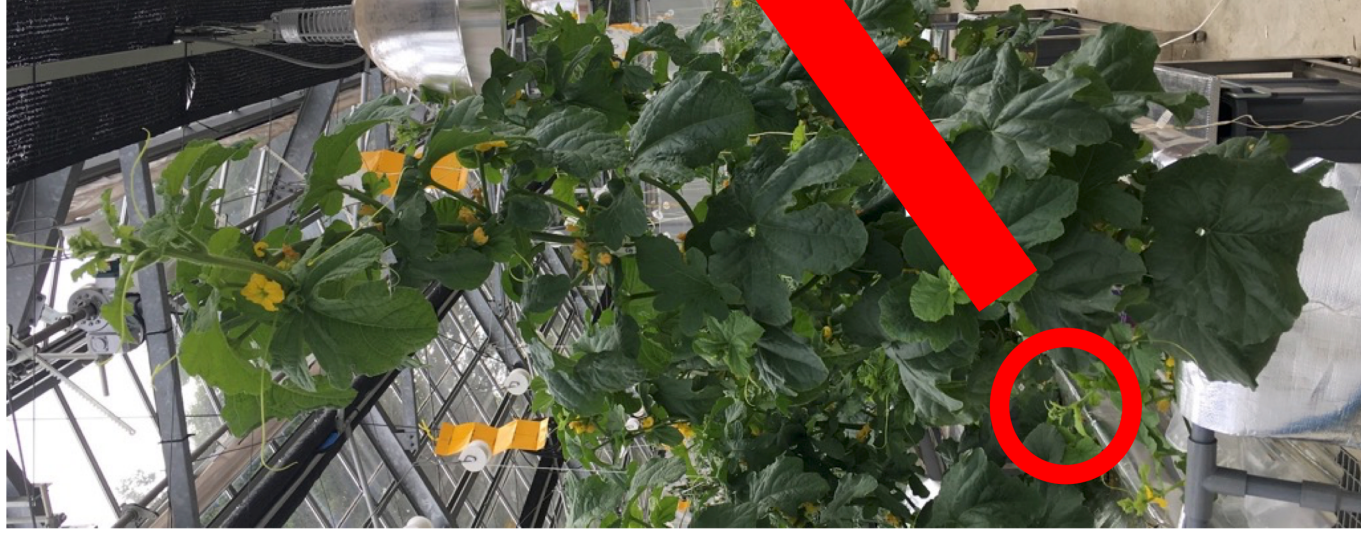

**10 young leaves**

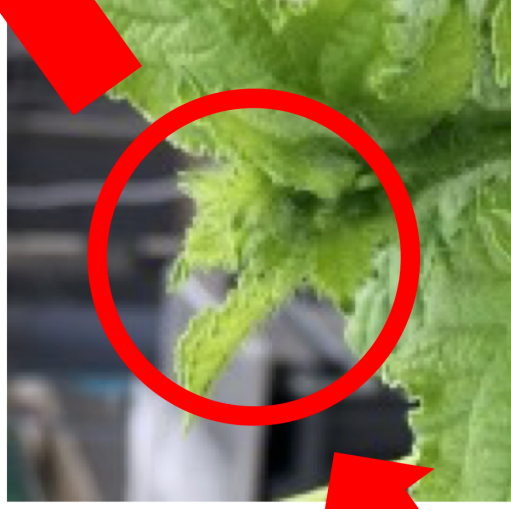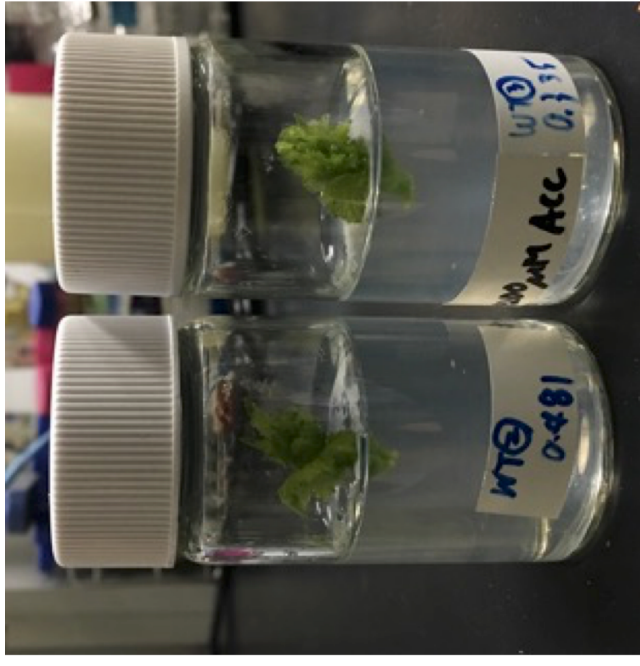

**200  $\mu$ M  
ACC**

**+**

**Incubation at 30°C for 24 hr**

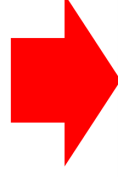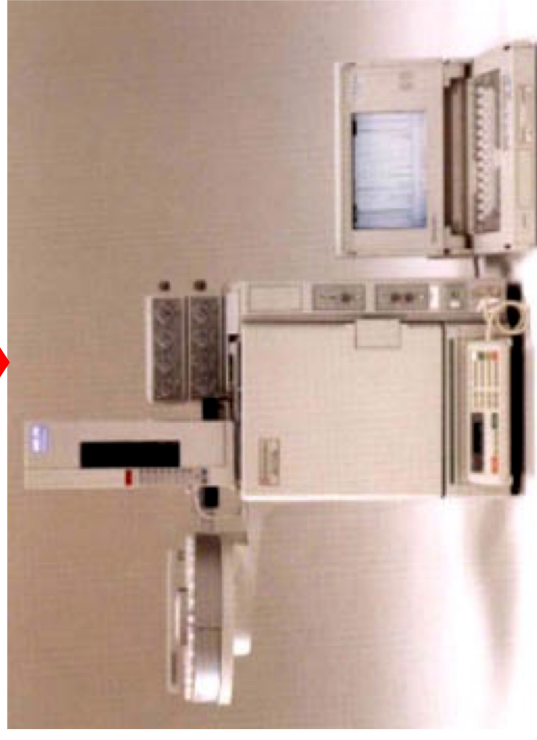

Supplement: Supplementary file 6 [file DataSheet5.PDF]
