# Supplementary material for: Targeted modification of CmACO1 by CRISPR/Cas9 extends the shelf-life of Cucumis melo var. reticulatus melon
Source: Front Genome Ed. 2023 May 25;5:1176125. doi: 10.3389/fgeed.2023.1176125 (PMC10249633; doi:10.3389/fgeed.2023.1176125)
Supplement: Supplementary file 5 [file DataSheet1.PDF]

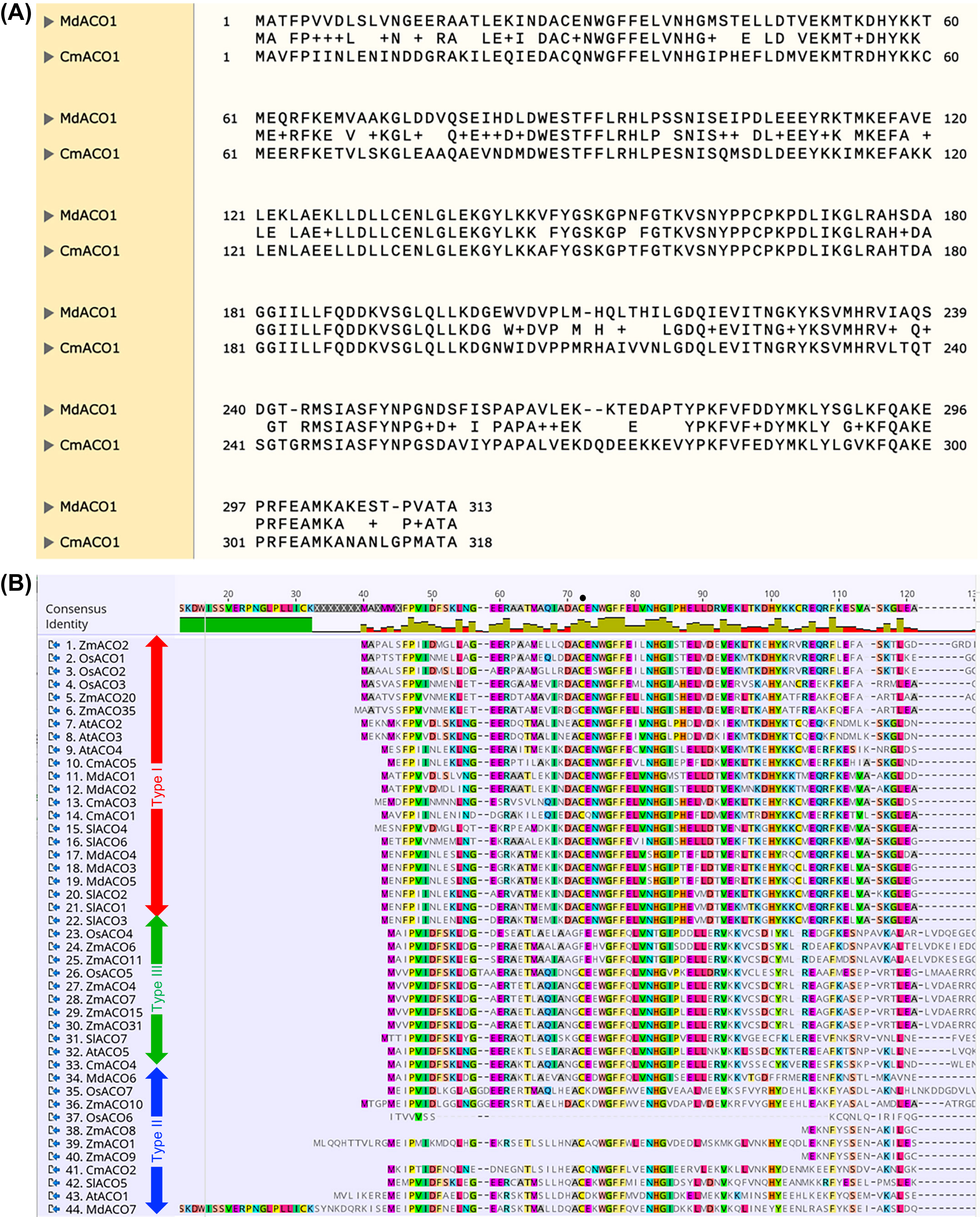
(A)
MdACO1 1 MATFPVVDLSLVNGEERAATLEKINDACENWGFFELVNHGMSTELLDTVEKMTKDHYKKT 60
MA FP+++L +N + RA LE+I DAC+NWGFFELVNHG+ E LD VEKMT+DHYKK
CmACO1 1 MAVFPIINLENINDDGRAKILEQIEDACQNWGFFELVNHGIPHEFLDMVEKMTRDHYKKC 60
MdACO1 61 MEQRFKEMVAAKGLDDVQSEIHDLDWESTFFLRHLPSSNISEIPDLEEEYRKTMKEFAVE 120
ME+RFKE V +KGL+ Q+E++D+DWESTFFLRHLP SNIS++ DL+EEY+K MKEFA +
CmACO1 61 MEERFKETVLSKGLEAAQAEVNDMDWESTFFLRHLPESNISQMSDLDEEYKKIMKEFAKK 120
MdACO1 121 LEKLAEKLLDLLCENLGLEKGYLKKVFYGSKGPNFGTKVSNYPPCPKPDLIKGLRAHSDA 180
LE LAE+LLDLLCENLGLEKGYLKK FYGSKGP FGTKVSNYPPCPKPDLIKGLRAH+DA
CmACO1 121 LENLAEELLDLLCENLGLEKGYLKKAFYGSKGPTFGTKVSNYPPCPKPDLIKGLRAHTDA 180
MdACO1 181 GGIILLFQDDKVSGLQLLKDGEWVDVPLM-HQLTHILGDQIEVITNGKYKSVMHRVIAQS 239
GGIILLFQDDKVSGLQLLKDG W+DVP M H + LGDQ+EVITNG+YKSVMHRV+ Q+
CmACO1 181 GGIILLFQDDKVSGLQLLKDGNWIDVPPMRHAIVVNLGDQLEVITNGRYKSVMHRVLTQT 240
MdACO1 240 DGT-RMSIASFYNPGNDSFISPAPAVLEK--KTEDAPTYPKFVFDDYMKLYSGLKFQAKE 296
GT RMSIASFYNPG+D+ I PAPA++EK E YPKFVF+DYMKLY G+KFQAKE
CmACO1 241 SGTGRMSIASFYNPGSDAVIYPAPALVEKDQDEEKKEVYPKFVFEDYMKLYLGVKFQAKE 300
MdACO1 297 PRFEAMKAKEST-PVATA 313
PRFEAMKA + P+ATA
CmACO1 301 PRFEAMKANANLGPMATA 318
(B)
Consensus
Identity
1. ZmACO2
2. OsACO1
3. OsACO2
4. OsACO3
5. ZmACO20
6. ZmACO35
7. AtACO2
8. AtACO3
9. AtACO4
10. CmACO5
11. MdACO1
12. MdACO2
13. CmACO3
14. CmACO1
15. SlACO4
16. SlACO6
17. MdACO4
18. MdACO3
19. MdACO5
20. SlACO2
21. SlACO1
22. SlACO3
23. OsACO4
24. ZmACO6
25. ZmACO11
26. OsACO5
27. ZmACO4
28. ZmACO7
29. ZmACO15
30. ZmACO31
31. SlACO7
32. AtACO5
33. CmACO4
34. MdACO6
35. OsACO7
36. ZmACO10
37. OsACO6
38. ZmACO8
39. ZmACO1
40. ZmACO9
41. CmACO2
42. SlACO5
43. AtACO1
44. MdACO7
Type I
Type III
Type II
